# Supplementary material for: Evidence and magnitude of the effects of meteorological changes on SARS-CoV-2 transmission
Source: PLoS One. 2021 Feb 17;16(2):e0246167. doi: 10.1371/journal.pone.0246167 (PMC7888632; doi:10.1371/journal.pone.0246167)
Supplement: S1 Table — (DOCX) [file pone.0246167.s001.docx]

S1 Table: The regression coefficients (abbreviated CT for coefficients of time) from the linear regression of the log of cumulative COVID-19 cases vs time (see Table 1) for each country were converted to their reciprocal values (CT^-1^).

| **Case Type** | **Dependent Variable** | **Independent Variables** | **Regression Coefficients** |  | **Adjusted R^2^** |
| --- | --- | --- | --- | --- | --- |
| Confirmed | CT | DP | -0.00098 | **** | 0.303 |
| Confirmed | CT^-1^ | DP | 0.308 | ******* | 0.492 |
| Confirmed | CT^-1^ | DP | 0.229 | **** | 0.584 |
|  |  | Days Cases (DC) | 0.151 | ** |  |
| Confirmed | CT^-1^ | DP | 0.266 | ***** | 0.599 |
|  |  | Days Cases (DC) | 0.134 | * |  |
|  |  | Land Area Per Capita (LAPC) | 23.942 | NS |  |
|  |  | MA | 0.098 | NS |  |

| **Case Type** | **Dependent Variable** | **Independent Variables** | **Regression Coefficients** |  | **Adjusted R^2^** |
| --- | --- | --- | --- | --- | --- |
| Deaths | CT | DP | -0.001 | * | 0.133 |
| Deaths | CT^-1^ | DP | 0.183 | ** | 0.195 |
| Deaths | CT^-1^ | DP | 0.165 | ** | 0.339 |
|  |  | Days Cases (DC) | 0.193 |  |  |
| Deaths | CT^-1^ | DP | 0.173 | ** | 0.315 |
|  |  | Days Cases (DC) | 0.204 | ** |  |
|  |  | Land Area Per Capita (LAPC) | 28.557 | NS |  |
|  |  | MA | -0.035 | NS |  |

| **Case Type** | **Dependent Variable** | **Independent Variables** | **Regression Coefficients** |  | **Adjusted R^2^** |
| --- | --- | --- | --- | --- | --- |
| Recovered | CT | DP | -0.0011 | *** | 0.271 |
| Recovered | CT^-1^ | DP | 0.3499 | ***** | 0.351 |
| Recovered | CT^-1^ | DP | 0.2344 | ** | 0.439 |
|  |  | Days Cases (DC) | 0.2017 | * |  |
| Recovered | CT^-1^ | DP | 0.2391 | * | 0.423 |
|  |  | Days Cases (DC) | 0.2105 | * |  |
|  |  | Land Area Per Capita (LAPC) | 16.1377 | NS |  |
|  |  | MA | -0.0643 | NS |  |

This was done because of an approximate proportionality of fitted CT with the standardized residuals when performing linear regression with Tmin MEDIAN [31]. A second regression was then performed using the CT^-1^ for each country as the dependent variable and independent variables consisting of median dewpoint [DP]) with or without regional demographic variables (i.e. land area and population median age). The total number of days for each country during which they had cases of COVID-19 between 1/22/20 and 4/6/20 (Days of Cases) was also used as a covariate. Also shown are the resulting estimated regression coefficients and the R^2^ for each regression. P values are displayed with progressively more stars for each 10-fold decrease in value according to this pattern: 0.05, 0.005, 0.0005, 0.00005, etc= *, **, ***, ****, etc. NS stands for Not Significant (P>0.05). All countries were included in the Confirmed Case Analysis. For Death Cases there were 4 countries not included for the following three reasons: 1) Insufficient data for Kuwait (3 days of 0 log cases) and Vietnam (no days of deaths), 2) Inconsistent and limited data for Slovakia (4 days 0 log cases, 10 days no reported cases, then 5 days 0 log cases followed by a single 0.3 log cases), 3) Outlier: Iceland. Recovered Cases had one exclusion: Outlier: Slovakia. Outliers were determined using the ROUT method of Graph Pad’s Prism software. Briefly, ROUT first fits a model to the data using a robust method where outliers have little impact. Then it uses a new outlier detection method, based on the false discovery rate, to decide which points are far enough from the prediction of the model to be called outliers [26].
