# Supplementary material for: Evidence and magnitude of the effects of meteorological changes on SARS-CoV-2 transmission
Source: PLoS One. 2021 Feb 17;16(2):e0246167. doi: 10.1371/journal.pone.0246167 (PMC7888632; doi:10.1371/journal.pone.0246167)
Supplement: S2 Table — (DOCX) [file pone.0246167.s002.docx]

S2 Table: Post-Hoc Analysis of Multivariable Regression for Temperature

| **Case Type** | **Dependent** | **Independent Variables** | **Regression**  **Coefficients** | **Adjusted R^2^** | **p value** | **p value x 3** | **p value x 3** |
| --- | --- | --- | --- | --- | --- | --- | --- |
|  | **Variable** |  |  |  |  |  |  |
| Confirmed | CT | Tmin | -0.000911 | 0.3 | **0.000023** | **0.000070** | ******** |
| Confirmed | CT^-1^ | Tmin | 0.275 | 0.449 | **0.000000061** | **0.000000182** | ********** |
| Confirmed | CT^-1^ | Tmin | 0.205 | 0.587 | **0.0000089** | **0.0000268** | ******** |
|  |  | Days Cases (DC) | 0.174 |  | **0.00016** | **0.00047** | ******* |
| Confirmed | CT^-1^ | Tmin | 0.247 | 0.61 | **0.0000015** | **0.0000044** | ********** |
|  |  | Days Cases (DC) | 0.15 |  | **0.0013** | **0.0039** | ****** |
|  |  | Land Area Per Capita (LAPC) | 21.244 |  | **0.14** | **0.43** | **NS** |
|  |  | MA | 0.151 |  | **0.11** | **0.33** | **NS** |
| **Case Type** | **Dependent Variable** | **Independent Variables** | **Regression**  **Coefficients** | **Adjusted R^2^** | **p value** | **p value x 3** | **p value x 3** |
| Deaths | CT | Tmin | -0.001 | 0.172 | **0.0024** | **0.0072** | ****** |
| Deaths | CT^-1^ | Tmin | 0.173 | 0.198 | **0.0011** | **0.0034** | ****** |
| Deaths | CT^-1^ | Tmin | 0.161 | 0.354 | **0.0008** | **0.0025** | ****** |
|  |  | Days Cases (DC) | 0.199 |  | **0.0014** | **0.0043** | ****** |
| Deaths | CT^-1^ | Tmin | 0.164 | 0.323 | **0.0015** | **0.0044** | ****** |
|  |  | Days Cases (DC) | 0.198 |  | **0.0029** | **0.0086** | ****** |
|  |  | Land Area Per Capita (LAPC) | -1.058 |  | **0.98** | **2.94** | **NS** |
|  |  | MA | 0.021 |  | **0.84** | **2.51** | **NS** |
| **Case Type** | **Dependent Variable** | **Independent Variables** | **Regression**  **Coefficients** | **Adjusted R^2^** | **p value** | **p value x 3** | **p value x 3** |
| Recovered | CT | Tmin | -0.00105 | 0.303 | **0.0000247** | **0.0000741** | ******** |
| Recovered | CT^-1^ | Tmin | 0.319 | 0.332 | **0.0000089** | **0.0000266** | ******** |
| Recovered | CT^-1^ | Tmin | 0.215 | 0.442 | **0.0025** | **0.0074** | ****** |
|  |  | Days Cases (DC) | 0.215 |  | **0.0024** | **0.0073** | ****** |
| Recovered | CT^-1^ | Tmin | 0.218 | 0.421 | **0.005** | **0.014** | ****** |
|  |  | Days Cases (DC) | 0.222 |  | **0.0060** | **0.018** | ***** |
|  |  | Land Area Per Capita (LAPC) | 13.446 |  | **0.57** | **1.70** | **NS** |
|  |  | MA | -0.0117 |  | **0.94** | **2.82** | **NS** |

The unit variable was country. At the until level, dependent and independent variables were evaluated. There were three correlated outcome measures: cases that were confirmed, deaths or recovered. For each of these three outcomes, an aggregate measure CT or CT^-1^ was calculated and then associated with multiple independent variables. Due to evaluating three outcomes in the same population, a multiple adjustment was required. A simple and conservative approach was used to inflate the observed p values by 3 (p*3) and compare at fixed alpha level [27, 28]. Also note that because CT and CT^-1^ are one-to-one transformations of one another, there was no need to adjust for using CT or CT^-1^ as an dependent variable.
