# Supplementary material for: Evidence and magnitude of the effects of meteorological changes on SARS-CoV-2 transmission
Source: PLoS One. 2021 Feb 17;16(2):e0246167. doi: 10.1371/journal.pone.0246167 (PMC7888632; doi:10.1371/journal.pone.0246167)
Supplement: S3 Table — (DOCX) [file pone.0246167.s003.docx]

S3 Table: Post-Hoc Analysis of Multiple Regression for Dewpoint

| **Case Type** | **Dependent Variable** | **Independent Variables** | **Regression**  **Coefficients** | **Adjusted R^2^** | **p value** | **p value x 3** | **p value x 3** |
| --- | --- | --- | --- | --- | --- | --- | --- |
| Confirmed | CT | DP | -0.00098 | 0.303 | **0.000020** | **0.000061** | ******** |
| Confirmed | CT^-1^ | DP | 0.308 | 0.492 | **0.0000000084** | **0.0000000251** | *********** |
| Confirmed | CT^-1^ | DP | 0.229 | 0.584 | **0.000011** | **0.000032** | ******** |
|  |  | Days Cases (DC) | 0.151 |  | **0.0014** | **0.0042** | ****** |
| Confirmed | CT^-1^ | DP | 0.266 | 0.599 | **0.0000027** | **0.0000080** | ********* |
|  |  | Days Cases (DC) | 0.134 |  | **0.006** | **0.017** | ***** |
|  |  | Land Area Per Capita (LAPC) | 23.942 |  | **0.11** | **0.32** |  |
|  |  | MA | 0.098 |  | **0.29** | **0.86** |  |
| **Case Type** | **Dependent Variable** | **Independent Variables** | **Regression**  **Coefficients** | **Adjusted R^2^** | **p value** | **p value x 3** | **p value x 3** |
| Deaths | CT | DP | -0.001 | 0.133 | **0.0074** | **0.0223** | ***** |
| Deaths | CT^-1^ | DP | 0.183 | 0.195 | **0.0013** | **0.0038** | ****** |
| Deaths | CT^-1^ | DP | 0.165 | 0.339 | **0.0014** | **0.0042** | ****** |
|  |  | Days Cases (DC) | 0.193 |  | **0.1930** | **0.5790** |  |
| Deaths | CT^-1^ | DP | 0.173 | 0.315 | **0.0019** | **0.0057** | ****** |
|  |  | Days Cases (DC) | 0.204 |  | **0.0024** | **0.0071** | ****** |
|  |  | Land Area Per Capita (LAPC) | 28.557 |  | **0.53** | **1.59** | **NS** |
|  |  | MA | -0.035 |  | **0.73** | **2.18** | **NS** |
| **Case Type** | **Dependent Variable** | **Independent Variables** | **Regression**  **Coefficients** | **Adjusted R^2^** | **p value** | **p value x 3** | **p value x 3** |
| Recovered | CT | DP | -0.0011 | 0.271 | **0.000075** | **0.00023** | ******* |
| Recovered | CT^-1^ | DP | 0.3499 | 0.351 | **0.0000044** | **0.000013** | ********* |
| Recovered | CT^-1^ | DP | 0.2344 | 0.439 | **0.0028** | **0.0084** | ****** |
|  |  | Days Cases (DC) | 0.2017 |  | **0.0058** | **0.017** | ***** |
| Recovered | CT^-1^ | DP | 0.2391 | 0.423 | **0.0056** | **0.017** | ***** |
|  |  | Days Cases (DC) | 0.2105 |  | **0.0067** | **0.020** | ***** |
|  |  | Land Area Per Capita (LAPC) | 16.1377 |  | **0.50** | **1.49** | **NS** |
|  |  | MA | -0.0643 |  | **0.66** | **1.98** | **NS** |

The unit variable was country. At the until level, dependent and independent variables were evaluated. There were three correlated outcome measures: cases that were confirmed, deaths or recovered. For each of these three outcomes, an aggregate measure CT or CT^-1^ was calculated and then associated with multiple independent variables. Due to evaluating three outcomes in the same population, a multiple adjustment was required. A simple and conservative approach has been used to compare observed p values (p) with adjusted alpha (=alpha/3) or inflate the observed p values by 3 (p*3) and compare at fixed alpha level [27, 28]. We adopted the latter approach. Also note that because CT and CT^-1^ are one-to-one transformations of one another, there was no need to adjust for using CT or CT^-1^ as an dependent variable.
