# Supplementary figures and images for: Evidence and magnitude of the effects of meteorological changes on SARS-CoV-2 transmission
Source: PLoS One. 2021 Feb 17;16(2):e0246167. doi: 10.1371/journal.pone.0246167 (PMC7888632; doi:10.1371/journal.pone.0246167)

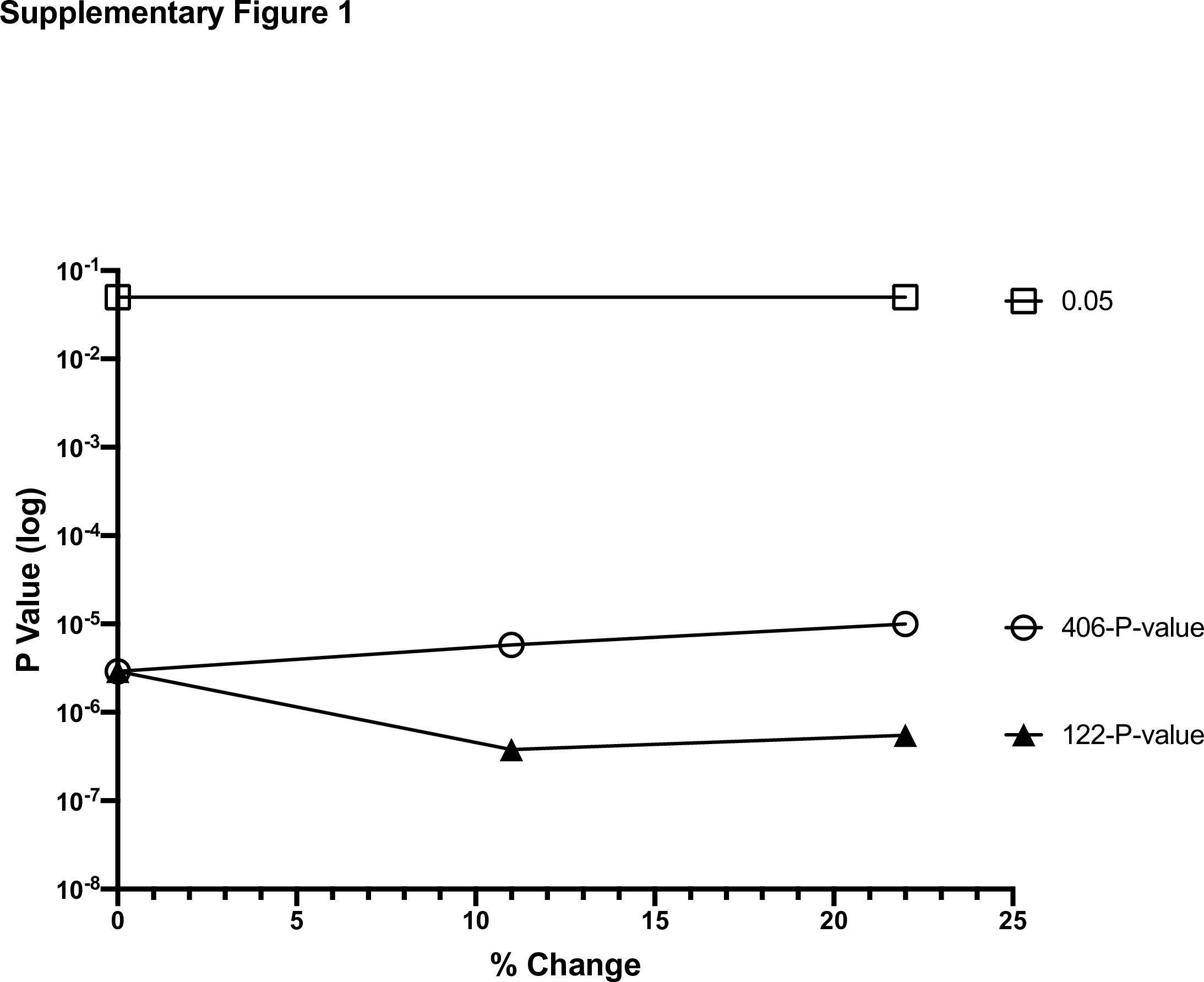

Supplement: S1 Fig — (TIF) [file pone.0246167.s004.tif]

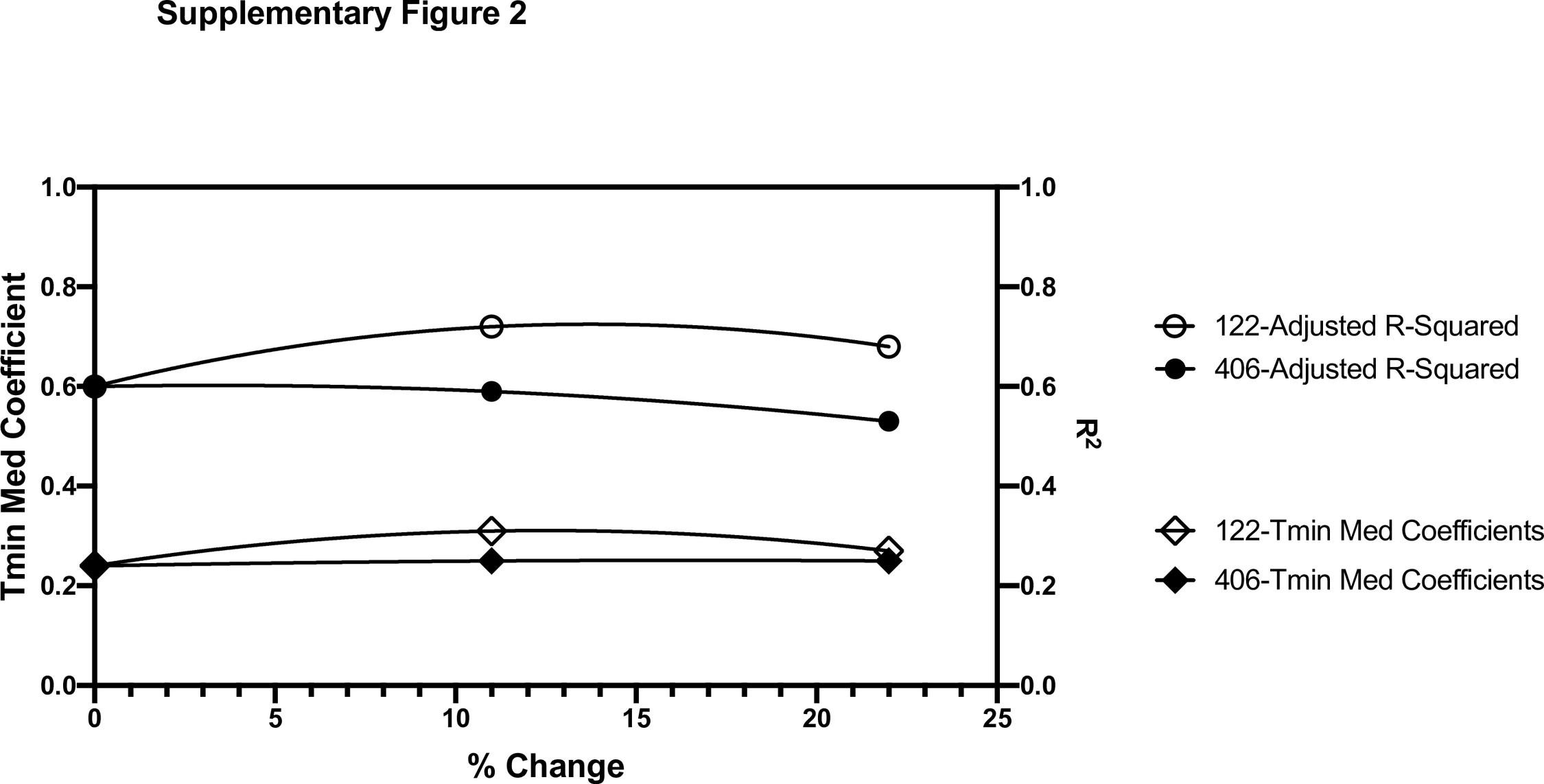

Supplement: S2 Fig — (TIF) [file pone.0246167.s005.tif]
