# Supplementary material for: Evidence and magnitude of the effects of meteorological changes on SARS-CoV-2 transmission
Source: PLoS One. 2021 Feb 17;16(2):e0246167. doi: 10.1371/journal.pone.0246167 (PMC7888632; doi:10.1371/journal.pone.0246167)
Supplement: Attachment — Submitted filename: Response to Reviewers 011321.docx [file pone.0246167.s008.docx]

Response to Editor Comments:

Additional Editor Comments (if provided):

The Authors have clarified most of the concern. However, it would be helpful to also include these explanations especially on the exclusion criteria in the manuscript, considering the following:
1. In the response, the authors provided valid reasons for the exclusion of China and Russia, especially the large variation in latitude and hence meteorological parameters.

In the manuscript, please state this explicitly as an exclusion criterion.

- The reasons for both China and Russia being excluded was made explicitly on lines 119-158.

Under-reporting of infections is likely common but the extent can vary greatly across countries. However, this would only affect the results if the level of under-reporting changed significantly over time within country.

- It would also affect the results if the level of underreporting did not change over time. This is the result of several logistical reasons that include the following:
- To approximate one scenario where the number of observed cases represent 2.3 % of the actual cases in Italy, we simply multiplied the number of cases by 2.3%--as depicted in the excel sheet shown below. [See below for the derivation of the 2.3% estimate of underreporting in Italy, taken from the data in Lau et al] (1).
  - Because the lead in number of cumulative daily cases are small initially (i.e. from 1/31/20 to 2/21/20) the reduction to 2.3% results in a fraction of cases or negative log values, as shown below. Because these are not actually obtainable, the initial lead in cases would not be seen and are therefore excluded from the observation and this would change the slope of the observed vs actual log of confirmed cases.
  - This is shown in the figure below titled “Italy Tmin vs Log Confirmed Cases (Actual vs 2.3%).” In fact, the slope changes by over 30% in these two scenarios.
- If on the other hand the reduction to observed cases being 2.3% of actual cases leads to just a shift of two identical curves save for the reduction by 97.7%--i.e. where the lead in is maintained but just delayed) then there are two further problems to contend with:
  - In this scenario, the initial rise in actual cases would have been shifted to a time prior to 1/22/20 when the JHU database first began collecting data. Although the data could be found from other sources, it would not have the same methodological consistency as all of the other countries included in the analysis. This would introduce a large potential variable, whereby 50 countries all had their data obtained through the same source (JHU) but one had a separate etiology. [It should be noted, as discussed previously in our second rebuttal, we explained why the initial rate of increase in confirmed cases is key to our approach in that it allows us to isolate the SARS-CoV-2 transmission rate during a period that is predominantly influenced by temperature prior to the implementation of non-pharmaceutical interventions.]
  - Also confounding would be the fact that in this scenario the shift in time to an earlier course of actual cases compared to observed, would change the temperature range over which the rise in cases appeared to have occurred. This discrepancy would affect the core of our approach and analysis. We devised a two-step process to investigate the relationship between Tmin and COVID-19 transmissibility,. In the first step, we examined linear regressions of the log of the daily cumulative COVID-19 cases versus time in sequential days (Figure 1). From this analysis we calculated the resulting regression coefficients (which we termed coefficients of time (CT) with units of log cases/day) for each country. In the second step, we investigated whether CT was associated with the median daily Tmin (Tmin_Med) in each country for the same time period that cases had been sampled. Thus, although vast underreporting of cases in Italy—even if constant over time—would not affect the CT for Italy, it would affect the appropriate Tmin_Med associated with that particular rate of transmission.
- In conclusion, vastly underreported cases in Italy would have an adverse effect on the data derived from including it in our analysis. We thus explained in our manuscript that the vast underreporting of cases in Italy was the basis of its exclusion. If the editor feels that our explanation that we provide here is essential to the readers understanding and appreciation of the manuscript, we can include in a supplementary section.

Please also note that Krantz et al and Lau et al. only gave consistent conclusion on under-reporting for the US and Spain, but very different for China (least under-reporting in Lau et al.), Italy and France. In any case, please provide evidence in the manuscript on the under-reporting / change in under-reporting over time if this is still the main reason for exclusion.

- The Lau report used two different methods of calculating the underreporting in the countries they included in their analysis: 1) estimating total COVID-19 cases and crude case-fatality risks (cCFR) (as shown in Table 3 in the paper), and 2) adjusting numbers to aCFR of Germany and South Korea (as shown in Figure 5 in the paper) (1).
- Here is a closer examination of the data presented from the first method taken from Table 3, with the right two columns containing additional calculations of the original data from the paper that we provided for clarity:

| Countries | Reported Cases | Estimated Cases | % Reported/Estimated | Fold Increase vs Italy |
| --- | --- | --- | --- | --- |
| China | 8.10E+04 | 1.50E+06 | 5% | 2 |
| South Korea | 8.30E+03 | 3.80E+04 | 22% | 9 |
| Japan | 8.30E+02 | 1.00E+04 | 8% | 4 |
| Italy | 2.80E+04 | 1.20E+06 | 2.3% | 1 |
| France | 6.60E+03 | 6.80E+04 | 10% | 4 |
| Spain | 9.20E+03 | 1.40E+05 | 7% | 3 |
| Iran | 1.50E+04 | 4.00E+05 | 4% | 2 |
| States | 3.50E+03 | 2.68E+04 | 13% | 6 |

- Thus, as can be seen in the table above, Italy had the lowest % reported cases (i.e. 2.3%), compared to total estimated cases, of the 8 countries examined. This was anywhere between 1/2 to 1/9^th^ the level of reporting of the other 7 countries included in the study.
- Thus, Lau provides evidence of the degree of underreporting in Italy and, as instructed, we included this reference and reasoning in our manuscript on lines 116-119.

2. Please reconsider if language is really a criterion for exclusion. I believe different languages were used in the included countries.

- All mention of language as the basis of exclusion was removed from the manuscript.
  - 1.	Lau H, Khosrawipour T, Kocbach P, Ichii H, Bania J, Khosrawipour V. Evaluating the massive underreporting and undertesting of COVID-19 cases in multiple global epicenters. Pulmonology. 2020.
